# Adaptation of ELISA detection of *Plasmodium falciparum* and *Plasmodium vivax* circumsporozoite proteins in mosquitoes to a multiplex bead-based immunoassay

**DOI:** 10.1186/s12936-021-03910-z

**Published:** 2021-09-23

**Authors:** Alice C. Sutcliffe, Seth R. Irish, Eric Rogier, Micaela Finney, Sarah Zohdy, Ellen M. Dotson

**Affiliations:** 1 The Centers for Disease Control and Prevention, Division of Parasitic Diseases and Malaria, Entomology Branch, Atlanta, GA USA; 2 President’s Malaria Initiative, Atlanta, GA USA; 3 Present Address: The Centers for Disease Control and Prevention, Division of Parasitic Diseases and Malaria, Malaria Branch, Atlanta, GA USA; 4 College of Science and Mathematics, Auburn University, Auburn, AL USA; 5 School of Forestry and Wildlife Sciences, Auburn University, Auburn, AL USA; 6 College of Veterinary Medicine, Auburn University, Auburn, AL USA; 7 Present Address: Entomology Department, College of Agriculture, Texas A&M University, College Station, TX USA

**Keywords:** *Plasmodium*, ELISA, Circumsporozoite, Sporozoite rate, Mosquito, Multiplex bead assay

## Abstract

**Background:**

*Plasmodium* spp. sporozoite rates in mosquitoes are used to better understand malaria transmission intensity, the relative importance of vector species and the impact of interventions. These rates are typically estimated using an enzyme-linked immunosorbent assay (ELISA) utilizing antibodies against the circumsporozoite protein of *Plasmodium falciparum, Plasmodium vivax* VK210 (*P. vivax*210) or *P. vivax* VK247 (*P. vivax*247), employing assays that were developed over three decades ago. The ELISA method requires a separate assay plate for each analyte tested and can be time consuming as well as requiring sample volumes not always available. The bead-based multiplex platform allows simultaneous measurement of multiple analytes and may improve the lower limit of detection for sporozoites.

**Methods:**

Recombinant positive controls for *P. falciparum*, *P. vivax*210 and *P. vivax*247 and previously developed circumsporozoite (cs) ELISA antibodies were used to optimize conditions for the circumsporozoite multiplex bead assay (csMBA) and to determine the detection range of the csMBA. After optimizing assay conditions, known amounts of sporozoites were used to determine the lower limit of detection for the csELISA and csMBA and alternate cut-off measures were applied to demonstrate how cut-off criteria can impact lower limits of detection. Sporozoite rates from 1275 mosquitoes collected in Madagascar and 255 mosquitoes collected in Guinea were estimated and compared using the established csELISA and newly optimized csMBA. All mosquitoes were tested (initial test), and those that were positive were retested (retest). When sufficient sample volume remained, an aliquot of homogenate was boiled and retested (boiled retest), to denature any heat-unstable cross-reactive proteins.

**Results:**

Following optimization of the csMBA, the lower limit of detection was 25 sporozoites per mosquito equivalent for *P. falciparum*, *P. vivax*210 and *P. vivax*247 whereas the lower limits of detection for csELISA were found to be 1400 sporozoites for *P. falciparum,* 425 for *P. vivax*210 and 1650 for *P. vivax*247. Combined sporozoite rates after re-testing of samples that initially tested positive for Madagascar mosquitoes by csELISA and csMBA were 1.4 and 10.3%, respectively, and for Guinea mosquitoes 2% by both assays. Boiling of samples followed by csMBA resulted in a decrease in the Madagascar sporozoite rate to 2.8–4.4% while the Guinea csMBA sporozoite rate remained at 2.0%. Using an alternative csMBA cut-off value of median fluorescence intensity (MFI) of 100 yielded a sporozoite rate after confirmational testing of 3.7% for Madagascar samples and 2.0% for Guinea samples. Whether using csMBA or csELISA, the following steps may help minimize false positives: specimens are appropriately stored and bisected anterior to the thorax-abdomen junction, aliquots of homogenate are boiled and retested following initial testing, and an appropriate cut-off value is determined.

**Conclusions:**

The csMBA is a cost-comparable and time saving alternative to the csELISA and may help eliminate false negatives due to a lower limit of detection, thus increasing sensitivity over the csELISA. The csMBA expands the potential analyses that can be done with a small volume of sample by allowing multiplex testing where analytes in addition to *P. falciparum*, *P. vivax*210 and *P. vivax*247 can be added following optimization.

**Supplementary Information:**

The online version contains supplementary material available at 10.1186/s12936-021-03910-z.

## Background

Methods for determining the presence of *Plasmodium* spp. sporozoites in the salivary glands of potential mosquito vectors are key to measuring the intensity of malaria transmission, characterizing vector species, and evaluating intervention methods. Historically, infective mosquitoes have been identified through dissection of mosquito salivary glands. This is a time-consuming method requiring freshly-killed mosquitoes and does not allow for parasite species determination [1]. In the 1980s and 1990s, enzyme-linked immunosorbent assays (ELISAs) were developed that detect the highly stable major surface protein of the sporozoite, the circumsporozoite (cs) protein. Unlike detection by microscopy, the csELISA is performed on the head-thorax of preserved mosquitoes, and many specimens can be evaluated at the same time on a 96-well assay plate using species-specific antibodies for *Plasmodium falciparum, Plasmodium vivax* VK210 (*P. vivax*210) or *P. vivax* VK247 (*P. vivax*247) [2–4]. Assaying only the mosquito head-thorax reduces the possibility of detecting circulating sporozoites that have not yet migrated to the salivary glands, as well as cs protein from oocysts in the midgut [5]. The csELISA is frequently cited as the “gold standard” for determining the infectivity of mosquitoes and remains widely-used even with the development of sensitive molecular tools, such as various types of polymerase chain reaction (PCR) assays [6, 7]. While PCR is becoming more accessible in limited-resource settings, it is relatively expensive per sample analysed and many of the described methods are not *Plasmodium* species-specific. Thus, sequencing is required for further elucidation, and analyses with field-collected mosquitoes may not perform well if DNA is degraded or is only present in insufficient amounts to be amplified. Additionally, DNA-based detection is not parasite stage-specific. Consequently, it may overestimate infective mosquitoes by detecting DNA from non-infective parasite stages.

While csELISA is not subject to these limitations, its utility is limited by the necessity to test for the *P. falciparum*, *P. vivax*210 and *P. vivax*247 antigens in separate assays, meaning every sample needs to be analysed three times. This is a time-, sample- and reagent-consuming process, and 96-well assay plates can be highly variable with respect to antibody binding propensity, well shape and plate size [8, 9]. Consequently, the sensitivity of the assay may degrade, or inconsistent results may occur. Due to the practical limitations of the csELISA and molecular detection assays, the development of less time-consuming assays that are sensitive and specific is necessary for more accurate estimation of sporozoite rates.

Here, the adaptation and optimization of the gold standard csELISA to a bead-based assay platform is described. This technology allows for simultaneous or “multiplex” detection of multiple targets and has been used primarily for high-throughput detection of cytokines [10], antibodies [11, 12], and antigens [13, 14]. In the present context, a bead-based assay was optimized to allow for concurrent detection of *P. falciparum*, *P. vivax*210 and *P. vivax*247 cs proteins.

## Methods

### Mosquito collection

Uninfected mosquitoes for use as controls were obtained 5–10 days post-blood feeding from a laboratory colony of *Anopheles gambiae* G3 (U.S. Centers for Disease Control and Prevention, Atlanta, GA, USA). Mosquitoes were frozen overnight and placed on Drierite® in a sealed container at room temperature (approximately 20 °C) until use in either the csELISA or circumsporozoite multiplex bead assay (csMBA).

Quantified sporozoites collected from dissected mosquito salivary glands were provided by Dr. Jetsumon Prachumsri (Entomology Department of USAMC-AFRIMS, Bangkok, Thailand). These were received on dry ice as either pellets or in a residual volume of 10 mM PBS and were stored at -80 °C until use.

Two sets of wild caught mosquitoes from Madagascar and Guinea were analysed by csELISA and csMBA. The set from Madagascar contained 1275 *Anopheles* spp. mosquitoes collected using CDC miniature light traps baited with field-produced CO_2_ made from a sugar-yeast-water mixture and was stored at ambient temperatures on Drierite until arriving at the CDC (Atlanta, GA, USA) following shipment. The other set contained 255 *An. gambiae *sensu lato (*s.l*.) and *Anopheles funestus s.l.* collected in human landing collections, pyrethrum spray catches, aspiration, and light traps in Guinea. These were transported and stored at ambient temperature in 70% ethanol until dissection at the CDC (Atlanta, GA, USA).

### Preparation and analysis of sporozoites and mosquitoes

Mosquito heads-thoraces were separated from legs, wings, and abdomens between the second and third legs when possible [15], using a scalpel. The dissected heads-thoraces from Madagascar and negative control mosquitoes were placed into individual 1.7 ml centrifuge tubes with 50 µl of csELISA grind buffer (0.5% w/v Casein, 0.05% IGEPAL® CA-630, 0.002% w/v phenol red in 10 mM PBS, pH 7.4). A pellet pestle attached to a handpiece with a collet adapter (H.44B; Foredom) and powered by a Foredom® GG series motor (Foredom, USA) was used to grind mosquitoes until no discernable body parts were visible. Using a pipette, 200 µl of grind buffer was expelled over the pellet pestle and eluate was collected in the centrifuge tube containing the mosquito homogenate, yielding a final sample volume of approximately 250 µl. The pestle was wiped with a tissue and rinsed twice with PBS-T (10 mM PBS, 0.05% Tween20) between use. If being tested within 24 h of preparation, samples were placed at 4 °C. If they were to be tested more than 24 h later, they were stored at − 20 °C. Following csELISA, the remaining homogenate was stored at − 80 °C until processed by csMBA.

csELISA and csMBA were performed at the same time for the set of mosquitoes collected in Guinea. The head-thorax of each of these mosquitoes as well as negative controls were dissected as described above and placed individually in 1.2 ml collection tubes (Qiagen; 19560) containing a single 5 mm stainless steel bead (Qiagen; 69989), arranged in 96-place tube racks. Tubes were left open for approximately one hour at room temperature (approximately 20 °C) to allow evaporation of residual ethanol and then were stored at − 20 °C until the day they were processed. On the day of processing, 100 µl of csELISA grind buffer was added to each tube and samples were homogenized using a Qiagen TissueLyser II (Qiagen; 85300). Tube racks were placed in adapter sets (Qiagen; 69984) and agitated twice at 30 Hz for 30 s, changing the orientation between each agitation. A brief centrifugation was performed to collect liquid from the sides of the tube and concentrate debris.

On the day of use, the quantified sporozoites were prepared in grinding buffer in two-fold serial dilutions starting with 12,800 sporozoites.

Madagascar and Guinea mosquito samples that initially tested positive (“initial test”) by csELISA or csMBA were retested (“retest”) using both assays to check results [16]. In addition, samples that initially tested positive were incubated in a thermal cycler (Bio-Rad T100) for 10 min at 100 °C and retested (“boiled retest”) using csMBA to denature any heat-unstable cross-reactive proteins [17] and check results.

### Positive controls and antibodies

Positive controls, unlabelled capture, and horseradish peroxidase (HRP)-labelled secondary antibodies were obtained through BEI Resources, NIAID, NIH (MRA-890, MRA-1028 K). The *P.* *falciparum* positive control antigen is derived from recombinant protein R32tet_32_ produced in *Escherichia coli* (Smith Kline and French Laboratories, USA) [18]. The *P. vivax*210 and *P. vivax*247 positive control antigen, PvCSPv1, is a recombinant fusion protein that includes cs regions of both *P. vivax*210 and *P. vivax*247 produced in *Pichia pastoris* (Protein Potential, USA) [19]. All antibodies and positive controls were received lyophilized and were rehydrated to the concentrations indicated in the kit instructions [20]. Antibodies used for csELISA assays were rehydrated in equal parts glycerol and reverse osmosis de-ionized water (RO/DI) water. Antibodies used for csMBA were rehydrated with RO/DI water only. Sufficient stock volume was prepared to maintain uniform testing across all csELISAs and csMBAs. csELISA antibodies and all positive controls were stored at -20 °C and csMBA antibodies were stored at 4 °C following bead coupling and biotinylation.

### csELISA protocol

The protocol for the csELISA was followed as described by BEI Resources [20]. Separate assay plates were used for *P. falciparum*, *P. vivax*210 and *P. vivax*247 and all incubations occurred under a dark cover at room temperature (approximately 20 °C). Ninety-six well microplates (Costar 2797) were incubated with 50 µl of capture antibody solution (*P. falciparum* = 4 µg/ml; *P. vivax*210 and *P. vivax*247 = 2 µg/ml in 10 mM PBS) for 30 min. Following removal of the capture antibody solution, wells were filled with 200 µl of blocking buffer (0.5% w/v Casein, 0.002% w/v phenol red in 10 mM PBS, pH 7.4) and incubated for 1 h. After blocking buffer was removed, 50 µl of mosquito homogenate was added to preassigned wells and incubated for 2 h. Following incubation, homogenates were removed from the wells and samples were added back into their original tubes. Wells were then washed twice with PBS-T and incubated with HRP-labelled detection antibody solution (1 µg/ml in blocking buffer) for 1 h. Following removal of the detection antibody solution, wells were washed three times with PBS-T and incubated for 30 min with 2,2'-azino-bis(3-ethylbenzothiazoline-6-sulphonic acid (ABTS) substrate solution (Seracare; 51 -0032). Absorbance values were measured using a plate reader (Molecular Devices; SpectraMax 340) at λ = 405 nm. Samples were considered “positive” if the absorbance value was greater than two times the average absorbance value of the negative control wells [20].

### Binding of capture antibodies to polystyrene beads and biotinylation

*P. falciparum*, *P. vivax*210 and *P. vivax*247 monoclonal antibodies (mAbs) were covalently bound to polystyrene Bio-Plex® COOH beads (Bio-Rad; 1715060XX), each with a different bead designation, using the Luminex® xMAP® Antibody Coupling Kit (Luminex; 40-50016) and following the manufacturer’s protocols. All incubations were carried out at room temperature (approximately 20 °C) using an auto-rotator at 30 rpm. Wash steps were carried out using Activation Buffer (Luminex; 11-15171), centrifugation for 1.5 min at 14,548 × *g* and vortexing at a low setting. Following removal of the bead diluent and two washes, carboxyl groups on the microsphere surface were activated by incubating the microspheres for 20 min with Activation Buffer, 1-Ethyl-3-(3-dimethylaminopropyl)carbodiimide (EDC) and N-hydroxysulfosuccinimide (sulfo-NHS). Two wash steps were performed before beads were incubated for 2 h with activation buffer and monoclonal antibody to allow carboxyl-to-antibody amine crosslinking. Finally, two washes were performed, and bead-coupled antibodies were resuspended in 1 ml of Wash Buffer per 1 ml of starting volume of beads (Luminex 11-251167) and stored at 4 °C. For assay optimization, antibody concentrations of 10, 20 and 40 µg/ml were prepared in 50 µl reactions. Following selection of the optimal coupling concentration, a sufficient volume of beads was coupled to supply all experiments.

The *P. falciparum*, *P. vivax*210 and *P. vivax*247 monoclonal antibodies were biotinylated using the ThermoScientific EZ-Link Micro Sulfo-NHS-Biotinylation Kit (ThermoScientific; 21217) according to the manufacturer’s protocol. For assay optimization, sulfo-NHS-biotin concentrations of 89.2, 178.4 and 267.9 µg per ml of each antibody (originally resuspended to 0.5 mg/ml) were prepared in 50 µl reactions. Following selection of optimal sulfo-NHS-biotin concentration, a sufficient volume of antibody was biotinylated for use across all experiments.

### csMBA protocol

The csMBA protocol was adapted from the Rogier et al*.* antigen detection assay [14]. Following single-plex reactions for assay optimization, the bead assay was performed in multiplex containing *P. falciparum*, *P. vivax*210 and *P. vivax*247 coupled beads and detection antibodies. Reagent diluent (0.45 µM filter-sterilized PBS-T, 0.5% BSA) was used to dilute bead-coupled antibody, biotinylated detection antibody and streptavidin–phycoerythrin (Invitrogen; 2866). In each step where reagent diluent was used, 50 µl of solution was applied per well. Incubations were carried out, protected from light, at room temperature (approximately 20 °C) using a plate shaker (IKA; MTS 2/4) at 900 rpm and three wash steps with PBS-T were performed between each incubation. For the assay, filter bottom plates (Millipore; MADVN6550) were pre-wetted with PBS-T and 50 µl (approximately 1250 beads) of coupled beads were added, washed three times, and incubated with 50 µl of sample for 1.5 h. After washing, wells were incubated with a 50 µl mixture of *P. falciparum*, *P. vivax*210 and *P. vivax*247 biotinylated detection antibodies for 45 min. After washing, wells had a 30-min incubation with 50 µl of streptavidin–phycoerythrin in reagent diluent (Invitrogen; 2866). Wells had a final incubation with reagent diluent for 30 min before resuspension in 100 µl of PBS for a brief (1–2 min) incubation and were then either analysed immediately using a Bio-Plex® 200 system (BioRad; 171000201) and Bio-Plex® Manager™ software v6.2 (Bio-Rad, USA) with a target of 50 beads per region or stored at 4 °C, protected from light, for up to 24 h. Stored plates were washed three times following removal of PBS, wells were resuspended in 100 µl of PBS using a brief (1–2 min) incubation and then analysed as described above [21, 22]. The Bio-Plex® 200 system detects emitted fluorescence from the microspheres and the biotinylated detection antibody. Bio-Plex® Manager™ software reports fluorescence as the median fluorescence intensity (MFI) and generates the mean MFI for replicates when applicable. Background (Bkgd) values calculated from sample wells containing csELISA grind buffer alone were subtracted from MFI values to report the final assay signal as MFI-Bkgd.

### Optimization of multiplex bead assay

Recombinant positive controls were used for the optimization of the csMBA. All dilutions were run in triplicate and replicate MFI values were averaged to obtain a mean value. Two-fold serial dilution series were made in blocking buffer, beginning with 100 pg/50 µl, 9100 pg/50 µl and 4550 pg/50 µl for *P. falciparum*, *P. vivax*210 and *P. vivax*247, respectively, based on positive control dilutions in the csELISA protocol [20]. All bead assays performed during the assay optimization were conducted in single-plex, and assay performance was confirmed in multiplex format following selection of the optimal bead-antibody, biotinylated-antibody and streptavidin–phycoerythrin combination.

Antibody coupled to beads (capture antibody), sulfo-NHS-biotin conjugated to antibody (detection antibody) and streptavidin–phycoerythrin concentrations were varied to determine which combination allowed for optimized detection across a wide range of positive control concentrations (see Additional file 1). Antibody was coupled to beads in concentrations of 1, 2.5, 5, 10, 20 and 40 mg antibody per ml in 50 µl reactions. Sulfo-NHS-biotin concentrations of 89.2, 178.4 and 267.9 µg per ml of each antibody (originally resuspended to 0.5 mg/ml) were conjugated in 50 µl reactions. Streptavidin–phycoerythrin diluted 1:100 in reagent diluent was used in the assessment of all combinations of capture and detection antibody concentrations. A selected combination of capture (10 µg mAb/ml beads) and detection antibody (267.9 ng sulfo-NHS-biotin/µl of resuspended mAb) was assessed with streptavidin–phycoerythrin diluted to 1:100, 1:200 and 1:333 in reagent diluent, based on concentrations evaluated by Rogier et al*.* [14]. All dilutions were run in triplicate and replicate MFI values were averaged to obtain a mean value. Following the selection of optimal capture and detection antibody conditions, a sufficient volume of antibody was coupled with beads and biotinylated for use across all experiments.

## Results

### Optimization of bead assay

**Fig. 1:**
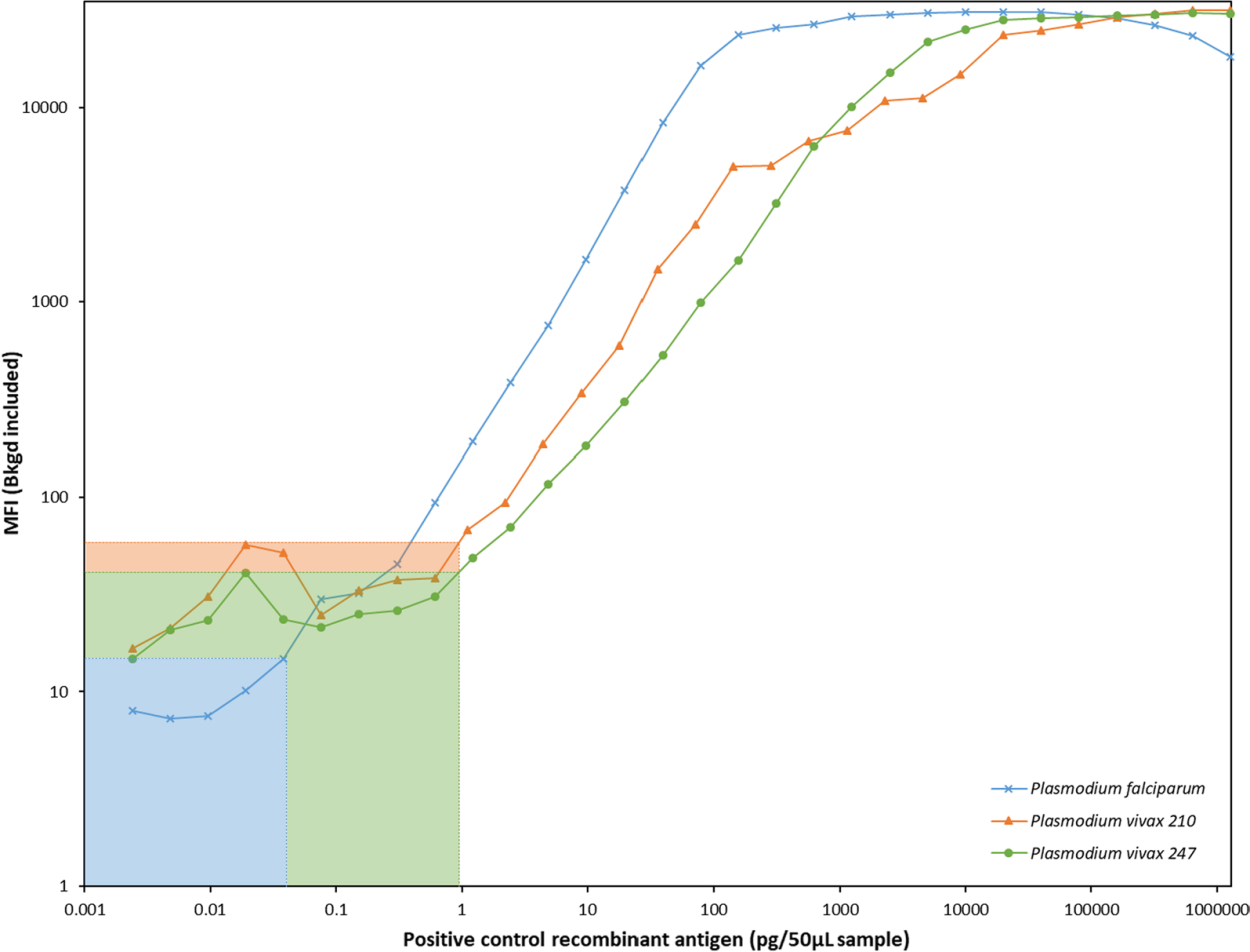
Median fluorescence intensity (including background) of serial dilutions of *Plasmodium falciparum*, *P. vivax*210 and *P. vivax*247 positive control antigen assayed with circumsporozoite multiplex-bead assay using optimized capture antibody, detection antibody and streptavidin–phycoerythrin conditions. The background fluorescence for wells containing blocking buffer only is indicated by the shaded regions

To simplify assay preparation, a standard concentration of each component (capture antibody = 10 µg mAb/ml beads, detection antibody = 267.9 ng sulfo-NHS-biotin/µl of resuspended mAb, streptavidin–phycoerythrin 1:333 dilution) was selected for *P. falciparum*, *P. vivax*210 and *P. vivax*247. This yielded a smooth, easily interpretable curve across the fluorescence detection range (3.5 logs) of the Luminex® array reader [23] (Fig. 1). The MFI upper range for all three analytes was greater than 3.0 × 10^4^ while background MFI for wells containing blocking buffer was ≤ 60. The range of cs protein positive control concentrations that could be detected between the background MFI and upper limit MFI was as follows: *P. falciparum* = 0.04 pg – 20 000 pg/50 µl, *P. vivax*210 = 1.1 pg – 1 300 000 pg/50 µl and *P. vivax*247 = 1.2 pg – 640 000 pg/50 µl (Table 1).

**Table 1:** Lower and upper limits of circumsporozoite (cs) multiplex-bead assay (MBA) median fluorescence intensity (MFI) values and detection of positive control for *Plasmodium falciparum*, *P. vivax*210 and *P. vivax*247

	Limits (MFI)		Positive control detection (pg/50µL)	
	Lower	Upper	Lower	Upper
*P. falciparum*	15	30 940	0.04	20 000
*P. vivax*210	59	31 556	1.1	1 300 000
*P. vivax*247	41	30 500	1.2	640 000

The MFI values for the lower limits of csMBA represent the background MFI contribution from blocking buffer only

### csELISA and csMBA comparison: lower limits of detection

**Fig. 2:**
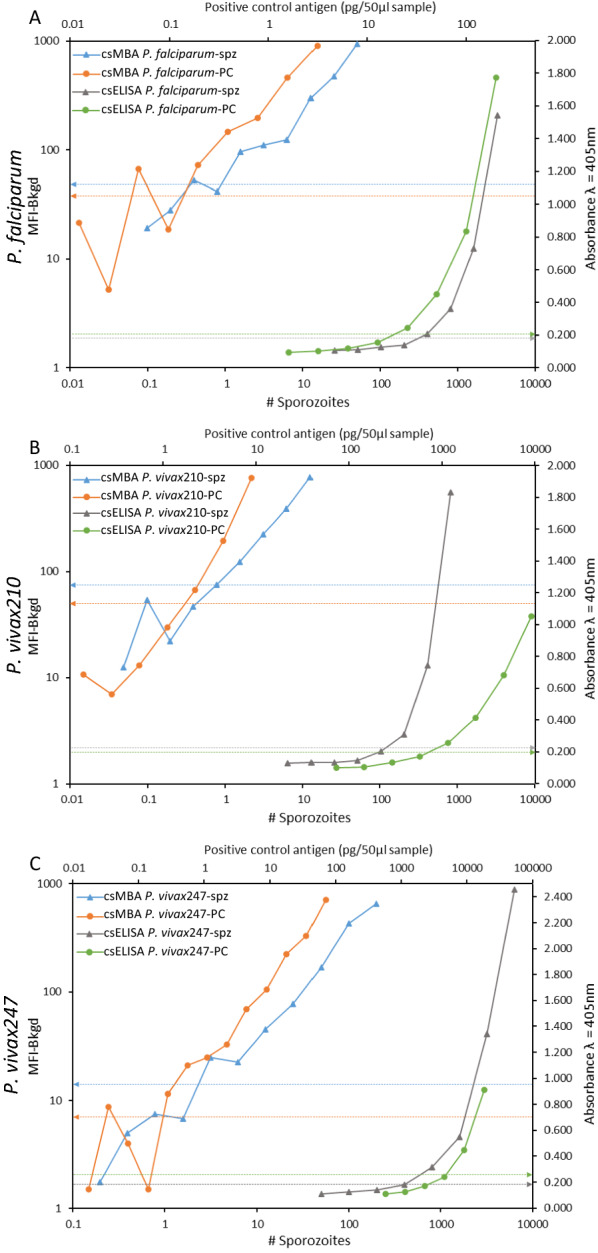
Lower range of detection for sporozoites (spz) and recombinant positive control (PC) for circumsporozoite (cs) enzyme-linked immunosorbent assay (ELISA) and cs multiplex-bead assay (MBA). When possible, the absorbance values at λ = 405 nm for four points above and below the cut-off value (indicated by dashed lines) were plotted for csELISA. For csMBA, data points with median fluorescence intensity values minus background (MFI-Bkgd) < 1000 were plotted, up to four points below the cut-off. **A** *Plasmodium falciparum*. **B** *P. vivax*210. **C** *P. vivax*247 csMBA

The lower limits of detection for csELISA and csMBA were determined using recombinant positive controls as well as quantified sporozoites (Fig. 2). For the csELISA, positives are typically defined as samples that have an absorbance value higher than two times the average absorbance values of the negative control samples [20, 24]. Using this criterion, the csELISA lower limit of sporozoite detection was 1400 sporozoites for *P. falciparum*, 425 sporozoites for *P. vivax*210 and 1650 sporozoites for *P. vivax*247 per mosquito equivalent. The per mosquito equivalent was calculated by multiplying results by five, as each 50 µl sample assayed represents approximately one fifth of a mosquito (each mosquito was homogenized in 250 µl of grind buffer). A similar cut-off value of two times the average MFI values of the negative control samples for csMBA yielded a lower limit of detection of approximately 25 sporozoites for *P. falciparum*, *P. vivax*210 and *P. vivax*247 per mosquito equivalent, representing a lower detection limit of 56x, 17x  and 66x, respectively, below those of the csELISA (Table 2). The lower limit of sporozoite detection for csMBA is reported here using alternative cut-off values calculated from the average of the negative control samples plus three standard deviations (Avg of negatives + 3SD) or a cut-off value of 100 (MFI-Bkgd > 100). Using the “Avg of negatives + 3SD” cut off, the lower limit of detection was 9, 10 and 32 sporozoites per mosquito equivalent and using the “MFI-Bkgd > 100 cut-off” gave a lower limit of detection of 12, 6 and 124 sporozoites per mosquito equivalent for *P. falciparum*, *P. vivax*210 and *P. vivax*247, respectively (Table 3).

**Table 2:** Lower limits of sporozoite detection for circumsporozoite (cs) enzyme-linked immunosorbent assay (ELISA) and cs multiplex-bead assay (MBA) extrapolated from best fit curve of twofold dilution series and rounded to nearest 25 for *P. falciparum, P. vivax210* and *P. vivax*247

	Lower limit of detection (# sporozoites/mosquito equivalent)			Fold-increase in csMBA sensitivity
	csELISA Previously reported	csELISA Current findings	csMBA	
*P. falciparum*	125 [2]	1400	25	56
*P. vivax*210	125 [4]	425	25	17
*P. vivax*247	250 [4]	1650	25	66

Fold-increase represents the csMBA limit of sporozoite detection compared to csELISA current findings. Previously published limits of sporozoite detection reported as sporozoites per 50 μl of mosquito homogenate, adjusted to "per mosquito equivalent" by multiplying by 5 are included

**Table 3:** Circumsporozoite (cs) multiplex-bead assay (MBA) lower limit of sporozoite detection for *Plasmodium falciparum, P. vivax*210 and *P. vivax*247 determined using three methods: **A**—traditional cut-off calculation method of two times the average (Avg) median fluorescence intensity minus background (MFI-Bkgd) of the negative control wells and two alternative methods: **B**—the average MFI-Bkgd of the negative control wells plus three standard deviations (SD) and **C**—MFI-Bkgd values greater than 100

Cut-off determination method	csMBA lower limit of detection (# sporozoites/mosquito equivalent)		
	*P. falciparum*	*P. vivax*210	*P. vivax*247
2 × Avg of negatives	5	4	13
Avg of negatives + 3SD	9	10	32
MFI > 100	12	6	124

### csELISA and csMBA comparison: wild caught mosquitoes

**Fig. 3:**
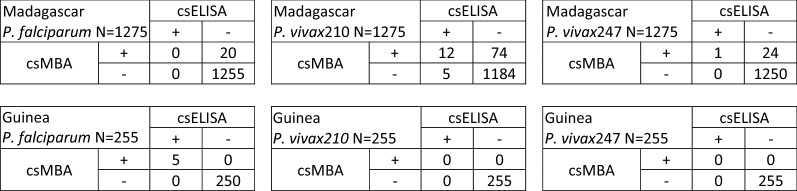
Two-by-two tables that reflect *Plasmodium falciparum, P. vivax*210 and *P. vivax*247 retest circumsporozoite (cs) enzyme-linked immunosorbent assay (ELISA) and cs multiplex-bead assay (MBA) results for Madagascar and Guinea

*Plasmodium falciparum*, *P. vivax*210 and *P. vivax*247 sporozoite rates derived from csMBA were higher than rates from csELISA for initial testing and retesting of the 1275 mosquitoes collected in Madagascar and the 255 mosquitoes collected in Guinea (Table 4). It was not possible to conduct boiled retesting by csELISA due to depleted sample volume. In the *P. falciparum* csMBA boiled retest of Madagascar samples, the positive control failed to elicit a fluorescence signal, thus invalidating results of those retests, and further testing was not possible due to depleted sample volume. The combined (*P. falciparum*, *P. vivax*210 and *P. vivax*247) Madagascar sporozoite rate determined by csELISA was initially 3.8% (49/1275) and after retesting, 1.4% (18/1275). Initial csMBA testing yielded a combined sporozoite rate of 15.8% (201/1275), retesting yielded 10.3% (131/1275) and boiled retesting (not including *P. falciparum* csMBA boiled retesting data due to issues described above) yielded 2.8% (36/1275). A possible alternative sporozoite rate approximation of 2.8–4.4% for boiled retesting was derived by combining the available boiled retest data with *P. falciparum* csMBA retest (without boiling) data. The combined Guinea sporozoite rate determined initially by csELISA was 2.4% (6/255) and 4.7% by csMBA (12/255). The sporozoite rates following retesting and boiled retesting (csMBA only) were both 2.0% (5/255). Figure 3 provides a detailed description of positive and negative results by csELISA and csMBA. The distribution of MFI-bgkd values for negative results are shown in Additional File 2.

**Table 4:** Summary of Madagascar and Guinea circumsporozoite (cs) enzyme-linked immunosorbent assay (ELISA) and cs multiplex bead assay (MBA) sporozoite rates for *Plasmodium falciparum, P. vivax*210 and *P. vivax*247

	Sporozoite rate % (count) Madagascar							
	*P. falciparum*		*P. vivax*210		*P. vivax*247		Combined	
	csELISA	csMBA	csELISA	csMBA	csELISA	csMBA	csELISA	csMBA
Madagascar n = 1275								
Initial Test	0 (0)	2.4% (31)	1.7% (22)	10.3% (131)	2.1% (27)	3.1% (39)	3.8% (49)	15.8% (201)
Retest	0 (0)	1.6% (20)	1.3% (17)	6.7% (86)	0.1% (1)	2.0% (25)	1.4% (18)	10.3% (131)
Boiled Retest	N/A	0 (n = 0)^a^	N/A	2.1% (27)	N/A	0.7% (9)	N/A	2.8% (36)^b^
Guinea n = 255								
Initial Test	2.4% (6)	3.5% (9)	0 (0)	1.2% (3)	0 (0)	0 (0)	2.4% (6)	4.7% (12)
Retest	2.0% (5)	2.0% (5)	0 (0)	0 (0)	0 (0)	0 (0)	2.0% (5)	2.0% (5)
Boiled Retest	N/A	2.0% (5)	N/A	0 (0)	N/A	0 (0)	N/A	2.0% (5)

^a^For these retests, positive controls failed and results could not be included

^b^Does not include boiled retest *P. falciparum* csMBA sporozoite rate

**Fig. 4:**
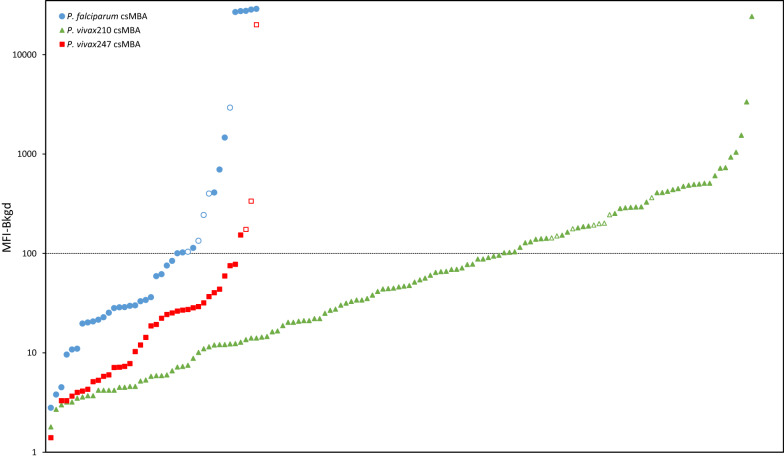
Madagascar and Guinea circumsporozoite (cs) multiplex-bead assay (MBA) median fluorescence intensity minus background (MFI-Bkgd) values for *Plasmodium falciparum*, *P. vivax*210 and *P. vivax*247, arranged from lowest to highest, for specimens that initially tested positive using a cutoff value determined by the average MFI of the negative mosquitoes multiplied by two. Data points below the dashed line have an MFI-Bkgd value of less than 100. Open markers indicate specimens with an initial MFI-Bkgd value > 100 that also had a retest MFI-Bkgd value > 100

The Guinea and Madagascar specimens that were initially determined to be positive using a cut-off value of two times the average MFI values of the negative control mosquitoes are shown in Fig. 4, which also demonstrates the outcome with the alternate cut-off value of MFI = 100. Comparing these cut-off criterion, initial testing identified 16 *vs* 40 (*P. falciparum*), 48 *vs* 134 (*P. vivax*210) and 3 *vs* 39 (*P. vivax*247) cs-positive samples. Retesting (without boiling) resulted in 11 (*P. falciparum*), 40 (*P. vivax*210) and 1 (*P. vivax*247) positives (Fig. 4). This gave a combined initial sporozoite rate and sporozoite rate after retesting for Madagascar of 4.6% (59/1275) and 3.7% (47/1275), respectively, and for Guinea, 3.1% (8/255) and 2.0% (5/255), respectively.

## Discussion

The conditions for the csMBA described here were optimized for reagent efficiency and allowed a detection range greater than 4.4 orders of magnitude for *P. falciparum*, *P. vivax*210 and *P. vivax*247 when using positive controls. The csMBA improved lower detection limits 56-fold, 17-fold and 66-fold for *P. falciparum*, *P. vivax*210 and *P. vivax*247, respectively, when compared to the csELISA and allowed for simultaneous detection of the three antigens. The process of multiplexing for these analytes allows a single csMBA run to generate the same amount of data as three csELISAs. In addition, the volume of antibody used in the csMBA was decreased by 80% for *P. falciparum* and 66% for *P. vivax*210 and *P. vivax*247 and the amount of sample used to test for *P. falciparum*, *P. vivax*210 and *P. vivax*247 decreased from 150 µl to 50 µl over the standard csELISA. The csMBA required approximately 3.5 h of hands-on preparation compared to approximately 5.5 h for the csELISA. Analysis steps for the csMBA performed using the Bio-Plex® 200 took approximately 1.75 h (passive time once analysis was initiated) while absorbance value acquisition for a csELISA plate took less than a minute with the SpectraMax 200. Thus, with the csMBA, instrument availability was the rate-limiting factor. Generally, the speed of MBA analysis varies based on settings within the software and how many beads are in the microplate wells but can be expected to take one to two hours. However, considering that the multiplex reader can analyse four 96-well csMBA assays in a typical workday, and each csMBA procedure simultaneously assays three sporozoite antigens, this is the equivalent of 12 csELISA 96-well plates (due to the single-plex nature of the csELISA). The availability of streamlining processes in the laboratory such as a plate washer, multichannel pipettes or a tissue homogenize, (like the TissueLyser) can enable more high-throughput sample processing. A workflow to prepare and analyse four plates by csMBA and the equivalent twelve plates by csELISA is shown in Additional file 3.

In addition to its greater efficiency, there is no “time penalty” for adding analytes to a bead-based assay, making this an ideal platform for expanding the targets to be detected. For example, inclusion of bead-bound antibody for the detection of additional parasite antigens could be accomplished without increasing assay preparation or run time. In contrast, with the csELISA, this would require preparation of an additional assay plate, representing 5.5 h, as well as additional sample depletion. In the context of mosquito analysis, one feasible application of this could be testing for other human *Plasmodium* spp., pending the development of sensitive and specific monoclonal antibodies, or host blood meal analysis, which, if analysed using csELISA, requires a separate assay plate (and 50 µl of sample) for each potential blood meal host and would be restricted due to higher required samples volumes.

The cost effectiveness of the csELISA as a laboratory method has made it a popular choice in resource-limited settings, where the malaria burden is often the highest. Initial costs of operationalizing a lab for csELISA can range from US$5-10 K and for csMBA from US$20-40 K. Using the materials and methods described here, and without factoring in personnel- or instrument maintenance-related costs, the amount (USD) to process a mosquito for a single analyte by csELISA is approximately $0.26 and for *P. falciparum*, *P. vivax*210 and *P. vivax*247 by csELISA is approximately $0.52. Comparable costs for csMBA analysis are $0.59 and $0.71, respectively (see Additional file 4). Thus, addition of each analyte represents a $0.13 increase by ELISA and $0.06 increase by MBA, therefore making MBA the more cost-efficient choice as the number of analytes being assayed increases. In the long-term, the time and cost-savings of MBA can offset the increased startup cost. Additional considerations such as decreased processing time and decreased detection limit make csMBA an attractive choice. The choice between ELISA and bead assay for cs-detection will depend on circumstances such as individual laboratory capacity, operating budget, processing throughput, and reagent and supplies availability.

Lower limits of detection for the csELISA previously reported during assay development were 125 sporozoites for *P. falciparum* and *P. vivax*210 and 250 sporozoites for *P. vivax*247 per mosquito equivalent [2, 4]. In contrast, lower limits of csELISA detection were found here to be (*P. falciparum* = 1400 sporozoite, *P. vivax*210 = 425 sporozoite and *P. vivax*247 = 1650 sporozoite per mosquito equivalent) and could be due to several factors. For example, differences in antibody lots, conjugation efficiency, antibody storage duration or the type of assay plate, due to differences in material, well shape and binding affinities used could all affect assay performance. The csMBA detected fewer than 25 sporozoite per mosquito equivalent, thus representing a significant improvement to detection sensitivity that could help minimize this problem. Repeatability of these findings was not conducted here and thus is a limitation of this study. This improved detection sensitivity means potentially more accurate identification of infective mosquitoes, thus reducing the possibility of false negatives and misleadingly low sporozoite rate estimations. The higher sporozoite rates determined by csMBA *versus* those by csELISA for mosquitoes collected in Madagascar may be due to increased assay sensitivity, that is, mosquitoes that did not contain enough sporozoite protein to be detected by csELISA but contained enough for detection by csMBA. This could be important for specimens with low levels of sporozoites and specimens where protein may have degraded due to poor storage conditions. For example, for *P. falciparum*, a range of 100 to 105,984 sporozoites was previously estimated using csELISA in salivary glands dissected from laboratory-infected *Anopheles stephensi* [25], and 130 to 245,760 and 82 to 77,270 sporozoites were found by microscopic examination of salivary glands dissected from naturally infected *An. gambiae* and *An. funestus*, respectively [26]. The average range of the number of *P. vivax* sporozoites from microscopic examination of salivary glands from batches of female mosquitoes fed on infected blood under laboratory conditions has been reported as 8.17-8347 [27]. In these examples, mosquitoes with lower sporozoite loads may be missed by the current csELISA but identified as positive using the csMBA. The two-by-two tables presented (Fig. 3) are difficult to interpret without knowing the infectivity status of each specimen. This can only be determined by dissection and microscopic examination of fresh specimens, which was not possible and thus, is a limitation in this study. In general, salivary gland dissections are difficult to execute as they are time consuming, time sensitive and require trained personnel [1]. The three mosquitoes from Guinea that initially showed positive results by *P. vivax*210 csMBA were determined to be negative after retesting and boiled retesting and were, therefore, not classified as positive (Table 4). Thus, additional analyses would be required to support *P. vivax* transmission in Guinea and data presented here is not intended to be used for that purpose.

The lower limit of detection observed with the csMBA when compared to the csELISA would suggest increased assay sensitivity. Therefore, it would be expected that analysis of specimens by csMBA would yield more cs-positive mosquitoes than the same analysis by csELISA. This was generally observed in this study, however, the Madagascar *P. vivax*210 results showed five samples positive by csELISA but negative by csMBA (Fig. 3). Without knowing the infectivity status of those mosquitoes, it is not possible to know the cause of this result. A possible explanation is that the csELISA and csMBA analyses were temporally separated for the Madagascar mosquitoes and thus, an unstable cross-reactive protein may have been detected by the csELISA that had degraded by the time csMBA analysis was conducted. Due to limited sample availability, it was not possible to boil homogenate and retest by csELISA. Ability to perform boiled retesting by csELISA may have helped to elucidate this observation and is thus a limitation of this study. A version of the csELISA protocol [16] available at the time of this study recommends initially testing all samples and retesting of positives (without boiling homogenate). This regimen creates potential to assign false positives if a cross-reactive protein is the cause for positive assay signal but also creates ambiguity if a sample tests positive initially but negative after retest. Retesting following boiling of homogenate may help to better explain and understand conflicting initial test and retest results.

It is widely accepted that the csELISA yields false positives, possibly due to detection of sporozoites circulating in haemolymph, detection of cs protein present in oocysts, or cross-reactivity of unknown protein thought to be present in livestock blood meals found in the mosquito digestive tract [28–30]. To address this, mosquitoes were bisected using a method shown to minimize unintended inclusion of oocysts, and homogenate was boiled prior to a portion of the retesting to denature any heat-unstable cross-reactive proteins [17]. Bisection, as described, was easier to accomplish for mosquitoes stored in ethanol (Guinea) than for dried specimens (Madagascar), which were brittle and crumbled under scalpel pressure. Mosquito storage conditions prior to analysis can also influence assay results, as protein can break down over time and mold or bacteria can grow if specimens are not properly desiccated. Thus, when possible, cold storage in 70% ethanol may help to improve protein preservation, minimize microbial growth, and enable proper dissection. Methods of storage and quality control measures were not systematically assessed in this study but may provide additional opportunity to further increase csELISA and csMBA sensitivity and specificity. An approach where sporozoite status is determined by microscopy and then those salivary glands are analysed by csELISA and csMBA would help to determine the true sensitivity and specificity of these laboratory assays, though even these types of studies can yield variable results [1, 5, 31]. Thus, proper bisection before testing specimens, followed by a regimen of boiled retesting of any initial positives is likely to increase confidence in the sensitivity of resulting sporozoite rates.

The assay signal cut-off value often used for immunoassays is the average of the negative controls plus three standard deviations [32], and anything above this cut-off value is considered “positive”. Minimal variation in the negative controls can reduce the cut-off value and potentially result in false positives. In the case of the csELISA, the current protocol prescribes using a cut-off value that is the average of the absorbance values of the negative controls, multiplied by two (without adjusting for background absorbance) [16, 20]. Depending on the contribution of the background, this can lead to artificially high cut-off values and can create the potential for false negatives. In addition, negative control mosquitoes are often obtained from a source independent of the test mosquitoes, which introduces variables such as storage conditions and environmental exposures, meaning these types of negative controls are not a true representation of collection negatives. Given these issues, an alternative method for determining positives is to choose a cut-off value that allows confident detection based on lower limits of detection. For example, with the csMBA, an MFI value of 100 (Table 3) would allow as few as 6 sporozoites per mosquito equivalent to be scored as a positive. Increasing this cut-off value would create potential for false negatives and conversely, decreasing it would create potential for false positives. To address this, boiling and retesting homogenate from samples above the cut-off will eliminate false positives that may be caused by heat-unstable cross-reactive proteins and strengthen the validity of the results. Further, to properly assess and compare results across multiple assay plates using an absolute cut-off, the background absorbance or fluorescence contribution specific to each assay plate (determined by assaying “grind buffer only” wells) should be subtracted from each sample reading.

Whether conducting csELISA or csMBA, a set of standardized quality control procedures can be established and conducted before a project begins and with each assay to increase confidence in results. For example, prior to starting a project, a sufficient volume of capture antibody, detection antibody and positive control can be prepared for use with all samples in a study. These preparations should be stored in aliquots to minimize freeze–thaw degradation and for protection from light to prevent photobleaching. A standard curve can then be generated in triplicate, using the prepared recombinant positive controls and antibodies. The standard curve data can be used to ensure a wide range of detection is possible and that the coefficient of variation (CV) between replicates is acceptable. Accuracy can be measured on each sample assay plate by including positive controls and comparing the absorbance-Bkgd or MFI-Bkgd values to those established with the standard curve. Outlier plates that do not fit the established acceptance criteria can be identified and samples on those plates rerun [33, 34]. While antibody and positive control preparation and storage were controlled in this study, accuracy between csELISA and csMBA sample plates was not compared and thus presents limitations that may make some of the Madagascar and Guinea sample data difficult to interpret and compare.

## Conclusions

The traditional ELISA used to estimate the number of infective mosquitoes for *P. falciparum*, *P. vivax*210 and *P. vivax*247 has been adapted and optimized for use as a bead-based assay. Antibody-based assays performed on the mosquito head-thorax allow for detection of protein only in the sporozoite stage, *versus* PCR, which amplifies DNA from any stage of parasite that may be present, not just that which is infective. The detection of infective mosquitoes is a pertinent measure for evaluating transmission intensity and the effectiveness of malaria control interventions. The cs protein is highly stable and therefore well suited for the methods by which field collected mosquitoes are stored and transported.

The development and validation of the described bead-based multiplex assay for detection of cs protein in mosquitoes using previously developed antibodies provides laboratories a cost-comparable and time saving alternative to the csELISA that will allow faster processing times and a lower limit of detection to estimate sporozoite rates, thus minimizing false negatives. A workflow has been described that can be applied to csELISAs or csMBAs to minimize false positives without adding significant processing time: mosquitoes should be properly stored prior to bisection anterior to the thorax-abdomen junction [15], and aliquots of homogenate found to be positive by initial testing should be boiled and retested to minimize false positives caused by heat-unstable cross-reactivity proteins [17]. This new platform also creates opportunity to assay for additional analytes, for example, other parasite or blood meal host proteins, without requiring additional processing time or required sample volume. The development of the csMBA is intended as an option to expand the toolbox of methods available for cs-detection, thus, as a potential alternative to the csELISA, when circumstances permit, rather than as a replacement for the widely used csELISA.

## Supplementary Information

**Additional file 1**: Circumsporozoite (cs) multiplex-bead assay (MBA) median fluorescence intensity minus background (MFI-Bkgd) values of recombinant positive control antigen with varying *Plasmodium falciparum*, *P. vivax*210 and *P. vivax*247 capture and detection antibody concentrations and streptavidin-phycoerythrin (RPE) concentrations for selection of optimal conditions.

**Additional file 2**: Distribution of multiplex-bead assay (MBA) median fluorescence intensity minus background (MFI-Bkgd) values of negative samples from Madagascar and Guinea for *Plasmodium falciparum*, *P. vivax*210 and *P. vivax*247. Values are log-transformed and are only shown for MFI-Bkgd values above zero. Those below zero are summarized in the top left corner of each histogram.

**Additional file 3**: Example of circumsporozoite (cs) enzyme-linked immunosorbent assay (ELISA) and cs multiplex-bead assay (MBA) workflow for assessing 384 samples with three analytes (*Plasmodium falciparum*, *P. vivax*210 and *P. vivax*247).

**Additional file 4**: Cost analysis of consumables used in the circumsporozoite (cs) enzyme-linked immunosorbent assay (ELISA) and cs multiplex-bead assay (MBA) for assessing three analytes (*Plasmodium falciparum*, *P. vivax*210 and *P. vivax*247) or a single analyte.

## Data Availability

All data is available upon reasonable request.

## Abbreviations

csELISA: Circumsporozoite enzyme-linked immunosorbent assay
csMBA: Circumsporozoite multiplex-bead assay
PBS: Phosphate buffered saline
PBS-T: Phosphate buffered saline-0.05% Tween 20
PCR: Polymerase chain reaction
MFI: Median fluorescence intensity
