# Supplementary material for: Adaptation of ELISA detection of Plasmodium falciparum and Plasmodium vivax circumsporozoite proteins in mosquitoes to a multiplex bead-based immunoassay
Source: Malar J. 2021 Sep 23;20:377. doi: 10.1186/s12936-021-03910-z (PMC8461957; doi:10.1186/s12936-021-03910-z)
Supplement: Supplementary file 1 — Additional file 1: Circumsporozoite (cs) multiplex-bead assay (MBA) median fluorescence intensity minus background (MFI-Bkgd) values of recombinant positive control antigen with varying Plasmodium falciparum, P. vivax210 and P. vivax247 capture and detection antibody concentrations and streptavidin-phycoerythrin (RPE) concentrations for selection of optimal conditions. [file 12936_2021_3910_MOESM1_ESM.pdf]

P. falciparum

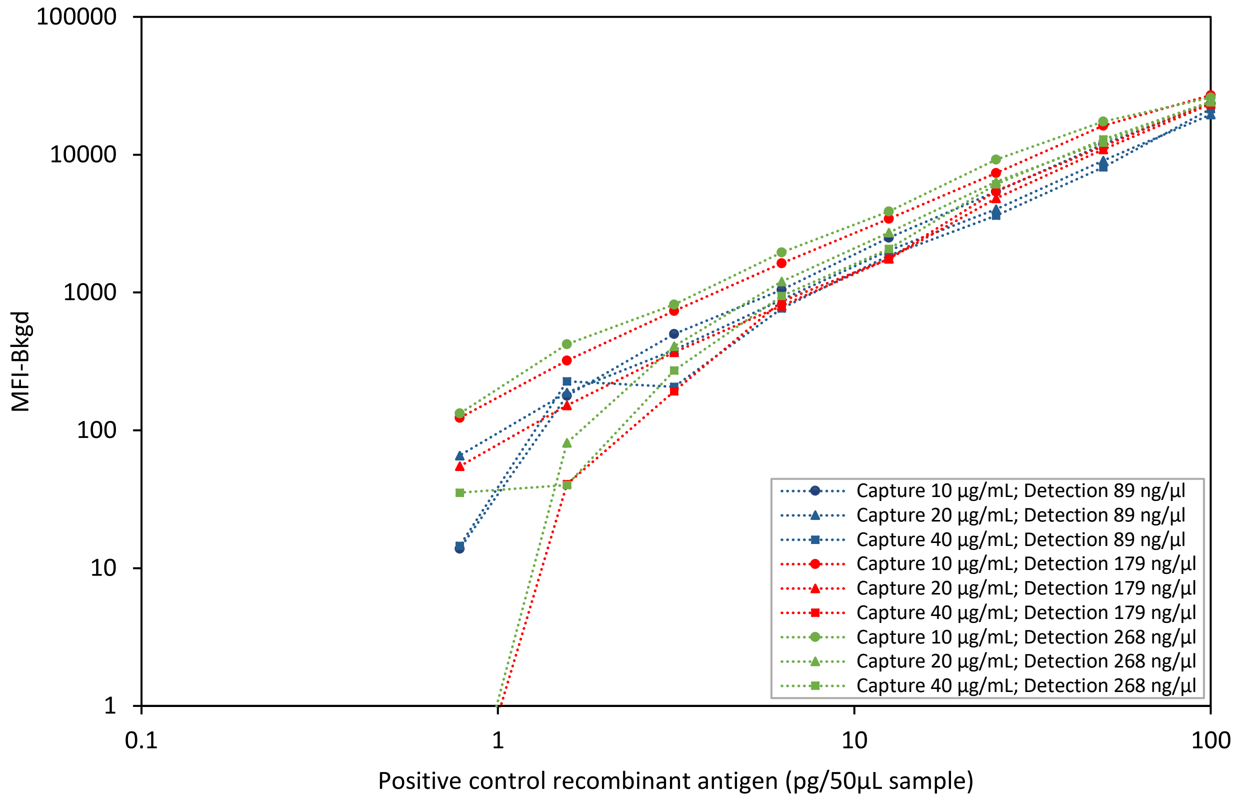

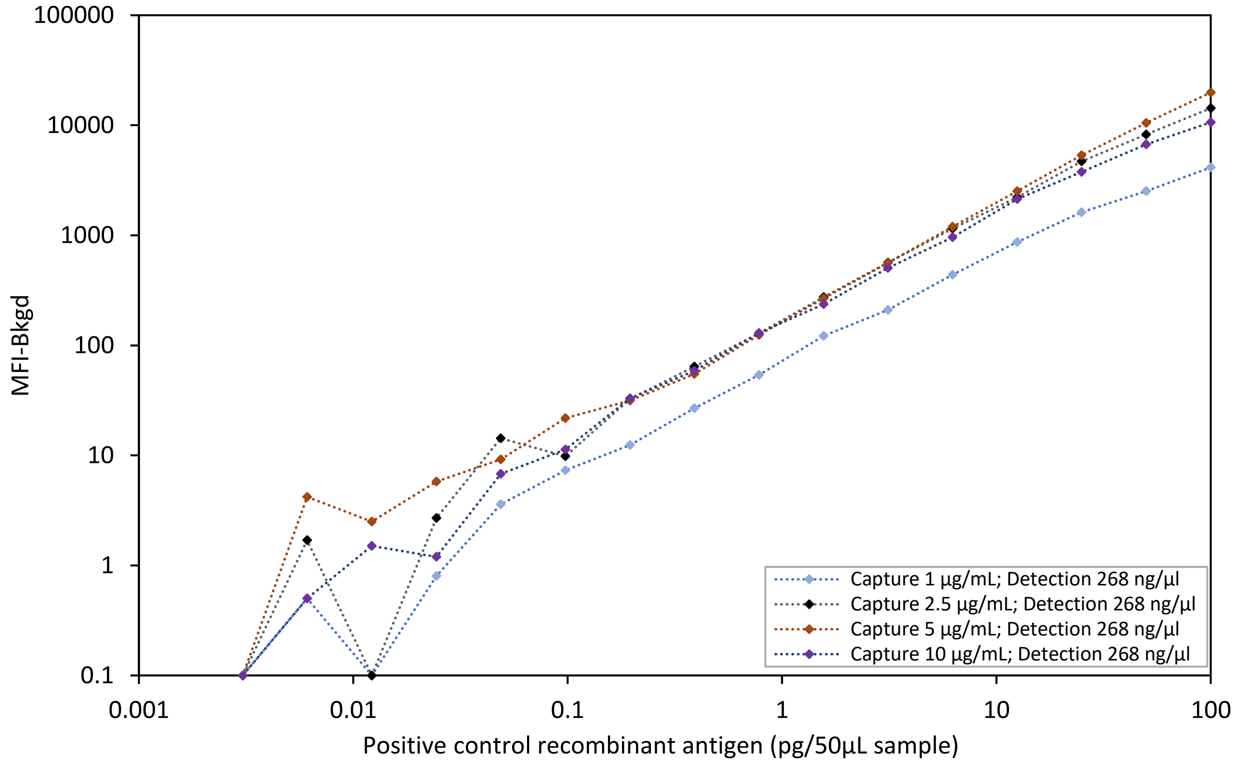

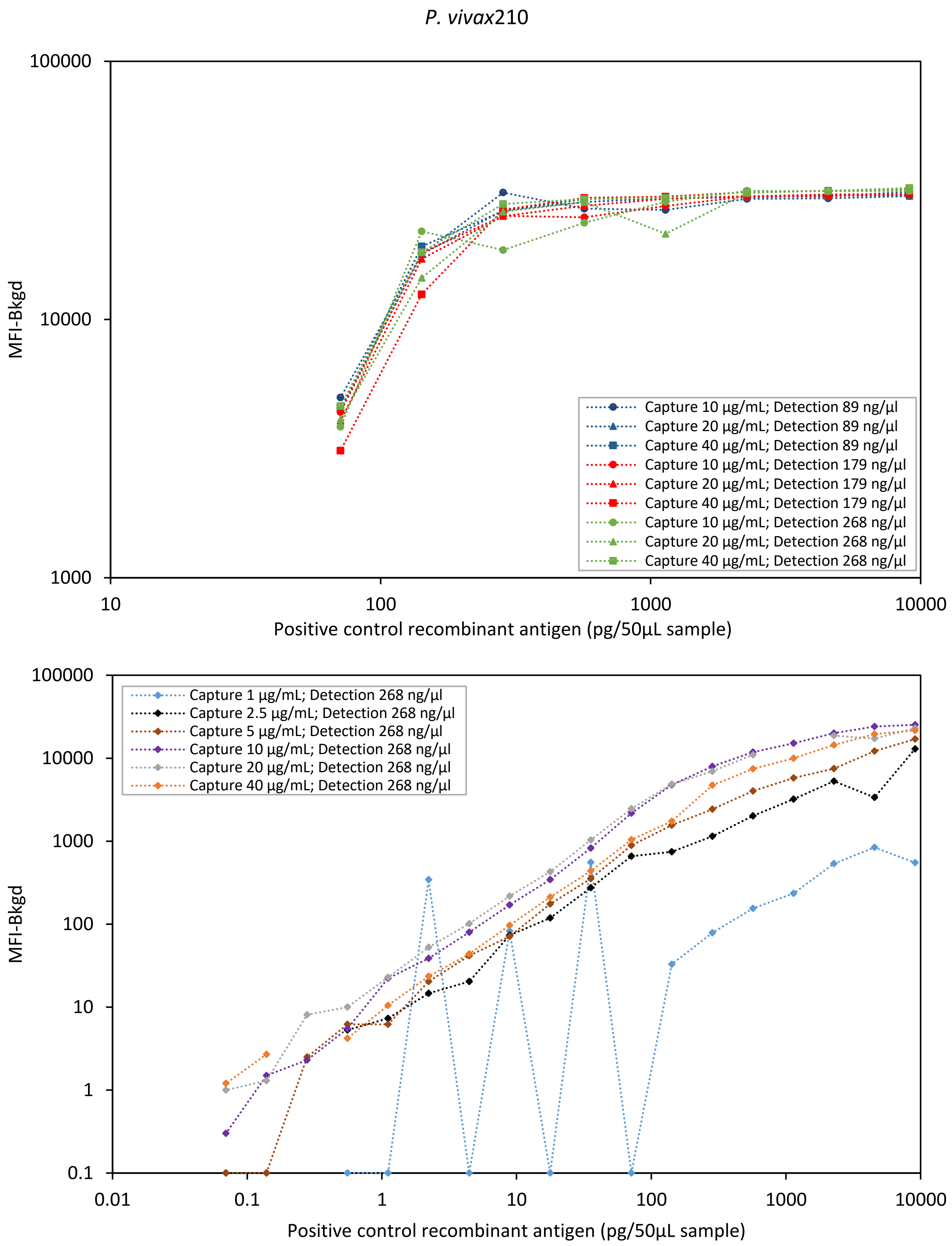
P. vivax210
100000
10000
1000
MFI-Bkgd
10
100
1000
10000
Positive control recombinant antigen (pg/50µL sample)
Capture 10 µg/mL; Detection 89 ng/µl
Capture 20 µg/mL; Detection 89 ng/µl
Capture 40 µg/mL; Detection 89 ng/µl
Capture 10 µg/mL; Detection 179 ng/µl
Capture 20 µg/mL; Detection 179 ng/µl
Capture 40 µg/mL; Detection 179 ng/µl
Capture 10 µg/mL; Detection 268 ng/µl
Capture 20 µg/mL; Detection 268 ng/µl
Capture 40 µg/mL; Detection 268 ng/µl
100000
10000
1000
100
10
1
0.1
MFI-Bkgd
0.01
0.1
1
10
100
1000
10000
Positive control recombinant antigen (pg/50µL sample)
Capture 1 µg/mL; Detection 268 ng/µl
Capture 2.5 µg/mL; Detection 268 ng/µl
Capture 5 µg/mL; Detection 268 ng/µl
Capture 10 µg/mL; Detection 268 ng/µl
Capture 20 µg/mL; Detection 268 ng/µl
Capture 40 µg/mL; Detection 268 ng/µl

*P. vivax*247

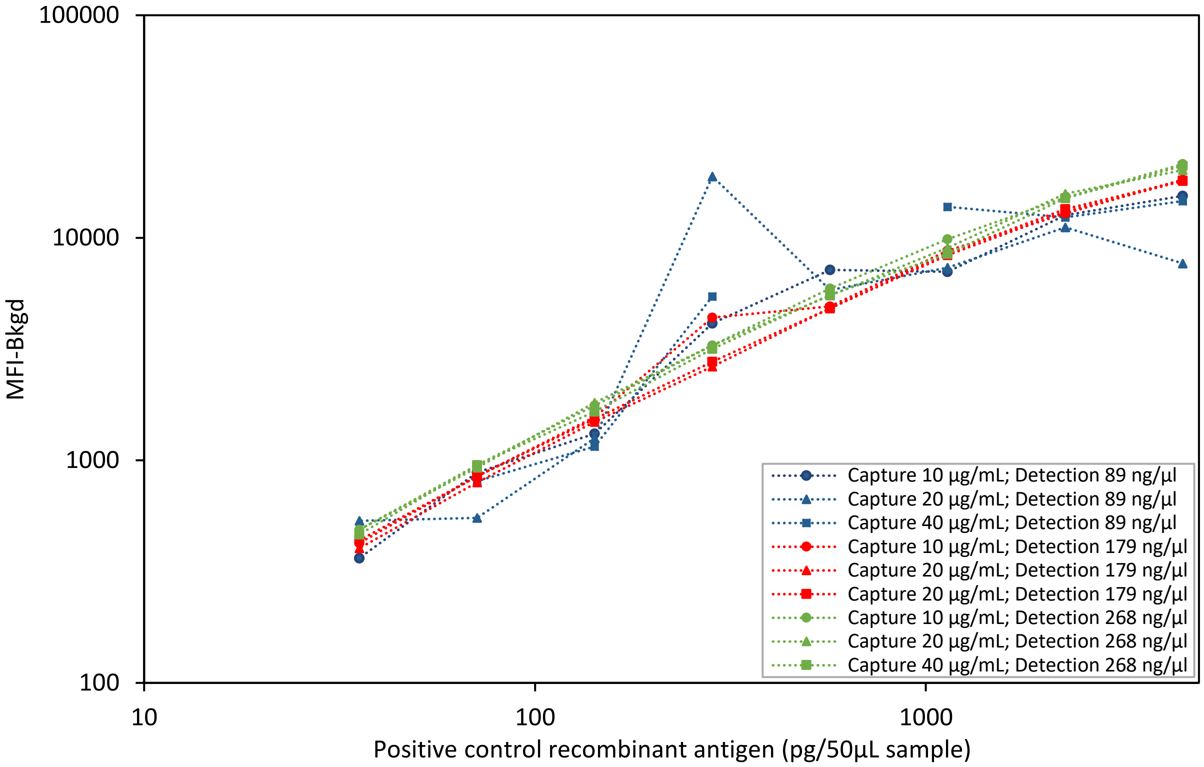
100000
10000
1000
100
MFI-Bkgd
10
100
1000
Positive control recombinant antigen (pg/50µL sample)
Capture 10 µg/mL; Detection 89 ng/µl
Capture 20 µg/mL; Detection 89 ng/µl
Capture 40 µg/mL; Detection 89 ng/µl
Capture 10 µg/mL; Detection 179 ng/µl
Capture 20 µg/mL; Detection 179 ng/µl
Capture 20 µg/mL; Detection 179 ng/µl
Capture 10 µg/mL; Detection 268 ng/µl
Capture 20 µg/mL; Detection 268 ng/µl
Capture 40 µg/mL; Detection 268 ng/µl

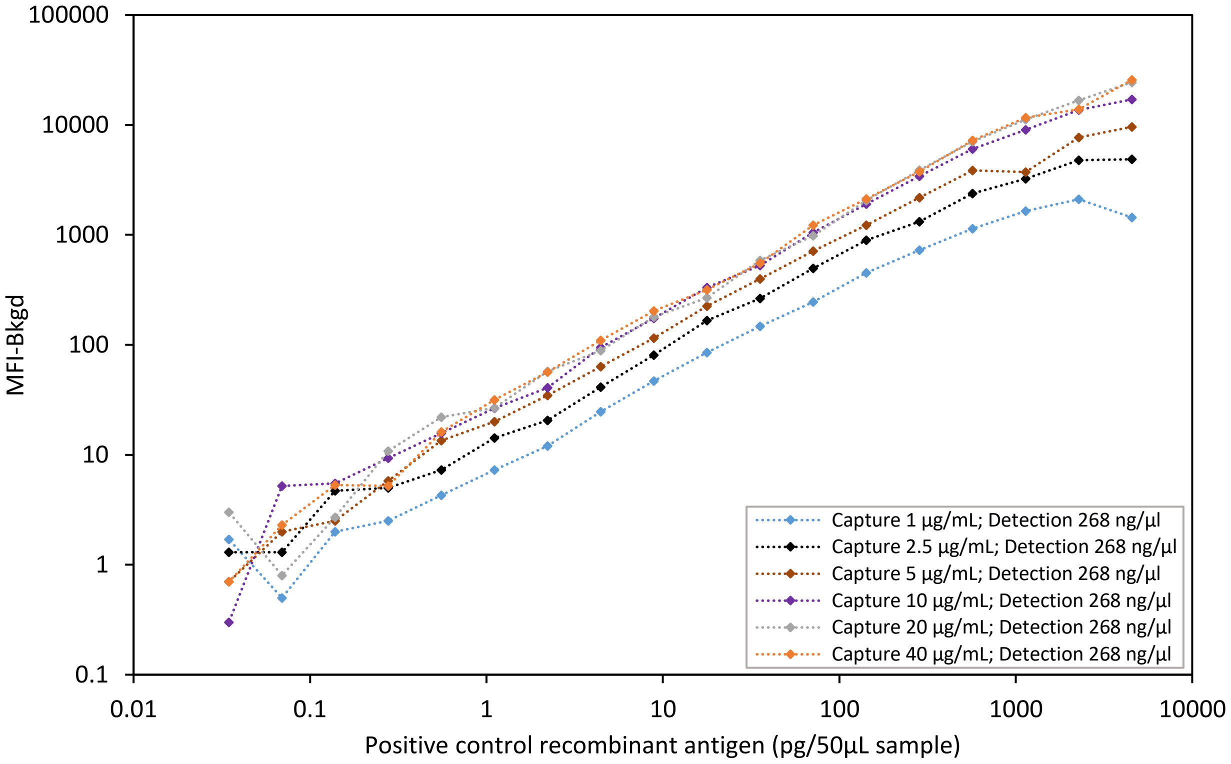
100000
10000
1000
100
10
1
0.1
MFI-Bkgd
0.01
0.1
1
10
100
1000
10000
Positive control recombinant antigen (pg/50µL sample)
Capture 1 µg/mL; Detection 268 ng/µl
Capture 2.5 µg/mL; Detection 268 ng/µl
Capture 5 µg/mL; Detection 268 ng/µl
Capture 10 µg/mL; Detection 268 ng/µl
Capture 20 µg/mL; Detection 268 ng/µl
Capture 40 µg/mL; Detection 268 ng/µl

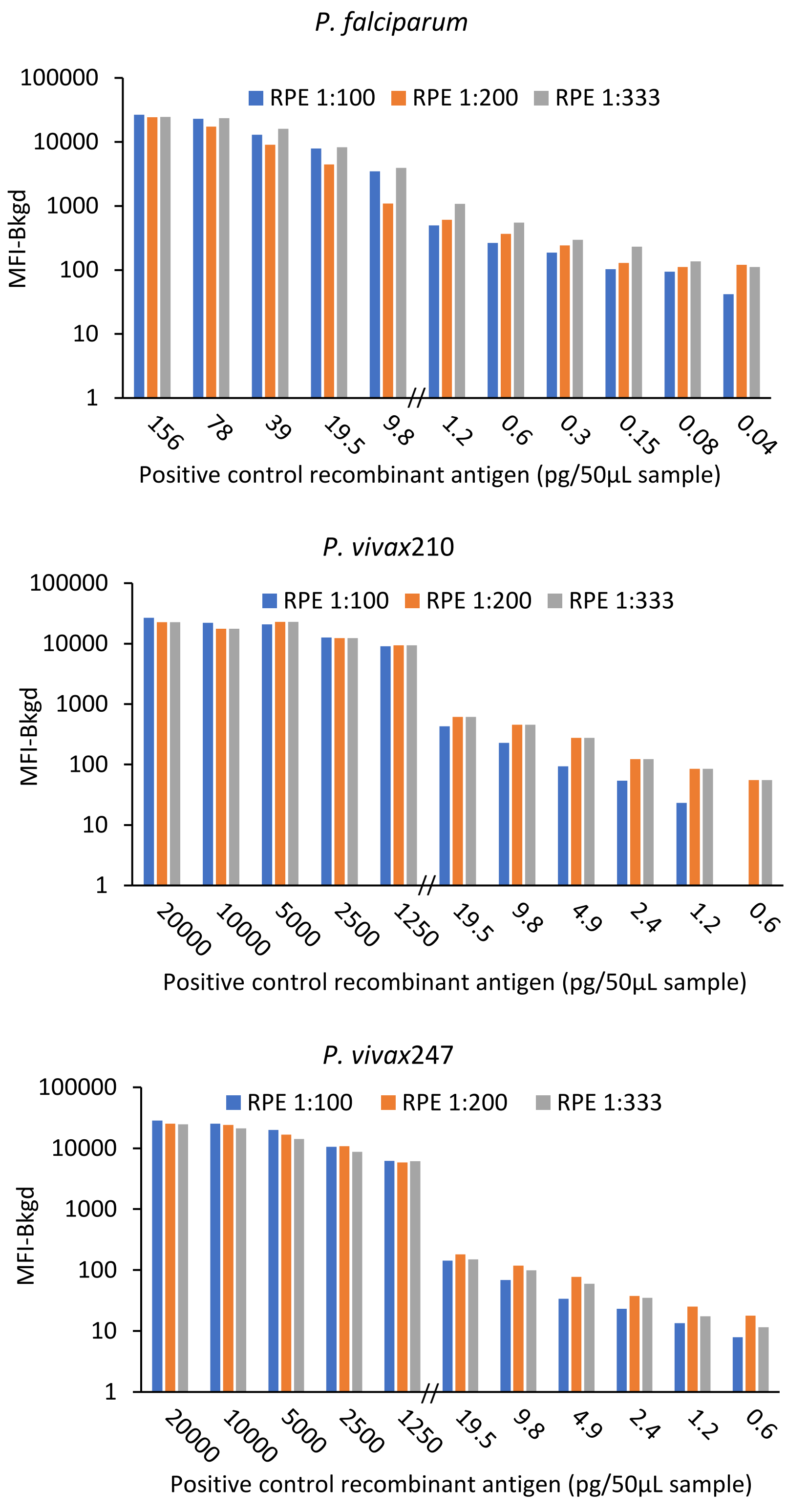

Additional file 1. Circumsporozoite (cs) multiplex-bead assay (MBA) median fluorescence intensity minus background (MFI-Bkgd) values of recombinant positive control antigen with varying *Plasmodium falciparum, P. vivax*210 and *P. vivax*247 capture and detection antibody concentrations and streptavidin-phycoerythrin (RPE) concentrations for selection of optimal conditions.
