# Supplementary material for: Adaptation of ELISA detection of Plasmodium falciparum and Plasmodium vivax circumsporozoite proteins in mosquitoes to a multiplex bead-based immunoassay
Source: Malar J. 2021 Sep 23;20:377. doi: 10.1186/s12936-021-03910-z (PMC8461957; doi:10.1186/s12936-021-03910-z)
Supplement: Supplementary file 2 — Additional file 2: Distribution of multiplex-bead assay (MBA) median fluorescence intensity minus background (MFI-Bkgd) values of negative samples from Madagascar and Guinea for Plasmodium falciparum, P. vivax210 and P. vivax247. Values are log-transformed and are only shown for MFI-Bkgd values above zero. Those below zero are summarized in the top left corner of each histogram. [file 12936_2021_3910_MOESM2_ESM.pdf]

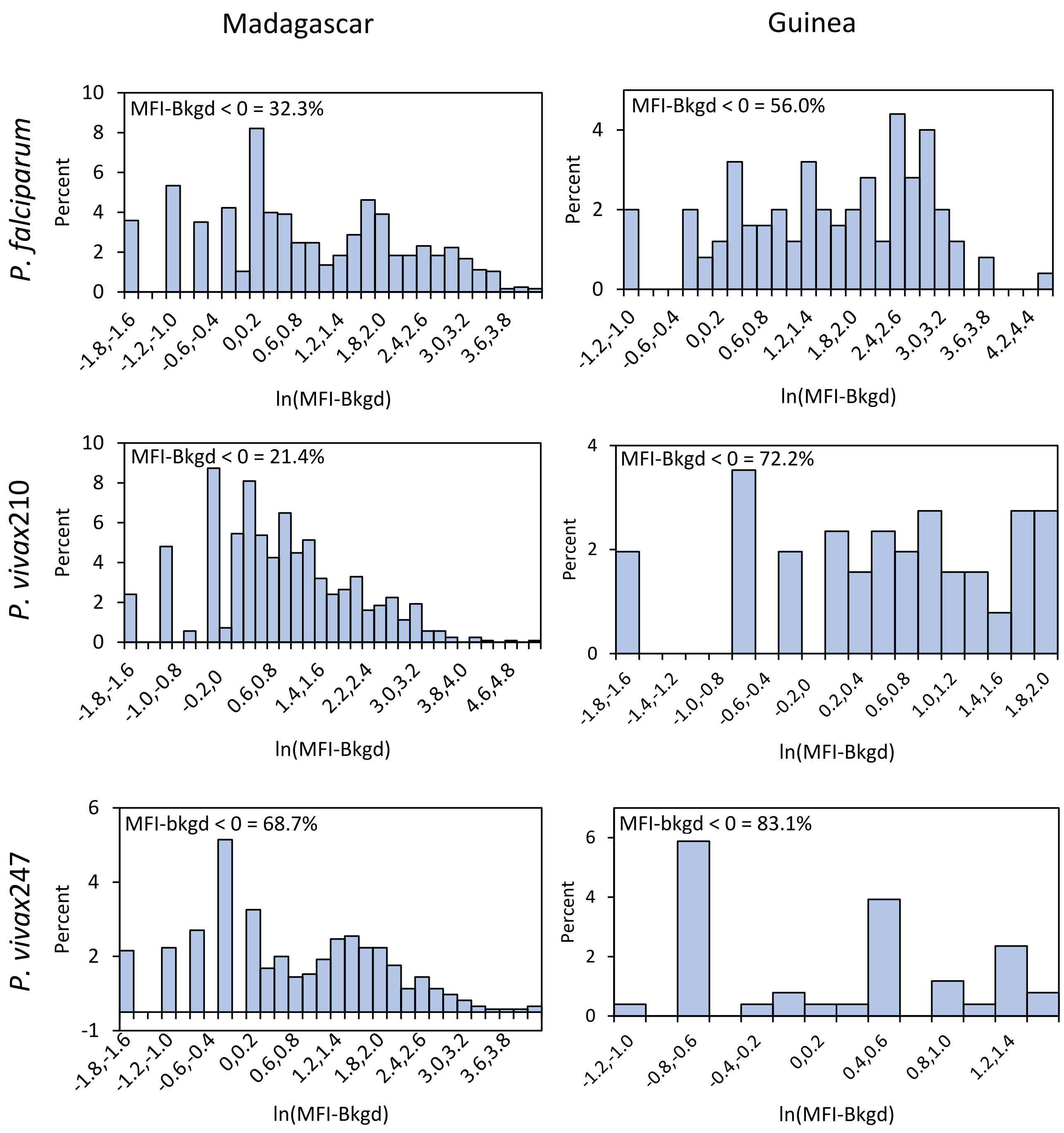

Additional file 4. Distribution of circumsporozoite multiplex-bead assay (csMBA) median fluorescence intensity minus background (MFI-Bkgd) values of negative samples from Madagascar and Guinea for *Plasmodium falciparum, P. vivax*210 and *P.vivax*247. Values are log-transformed and are only shown for MFI-Bkgd values above zero. Those below zero are summarized in the top-left corner of each histogram.
