# Supplementary material for: Adaptation of ELISA detection of Plasmodium falciparum and Plasmodium vivax circumsporozoite proteins in mosquitoes to a multiplex bead-based immunoassay
Source: Malar J. 2021 Sep 23;20:377. doi: 10.1186/s12936-021-03910-z (PMC8461957; doi:10.1186/s12936-021-03910-z)
Supplement: Supplementary file 3 — Additional file 3: Example of circumsporozoite (cs) enzyme-linked immunosorbent assay (ELISA) and cs multiplex-bead assay (MBA) workflow for assessing 384 samples with three analytes (Plasmodium falciparum, P. vivax210 and P. vivax247). [file 12936_2021_3910_MOESM3_ESM.pdf]

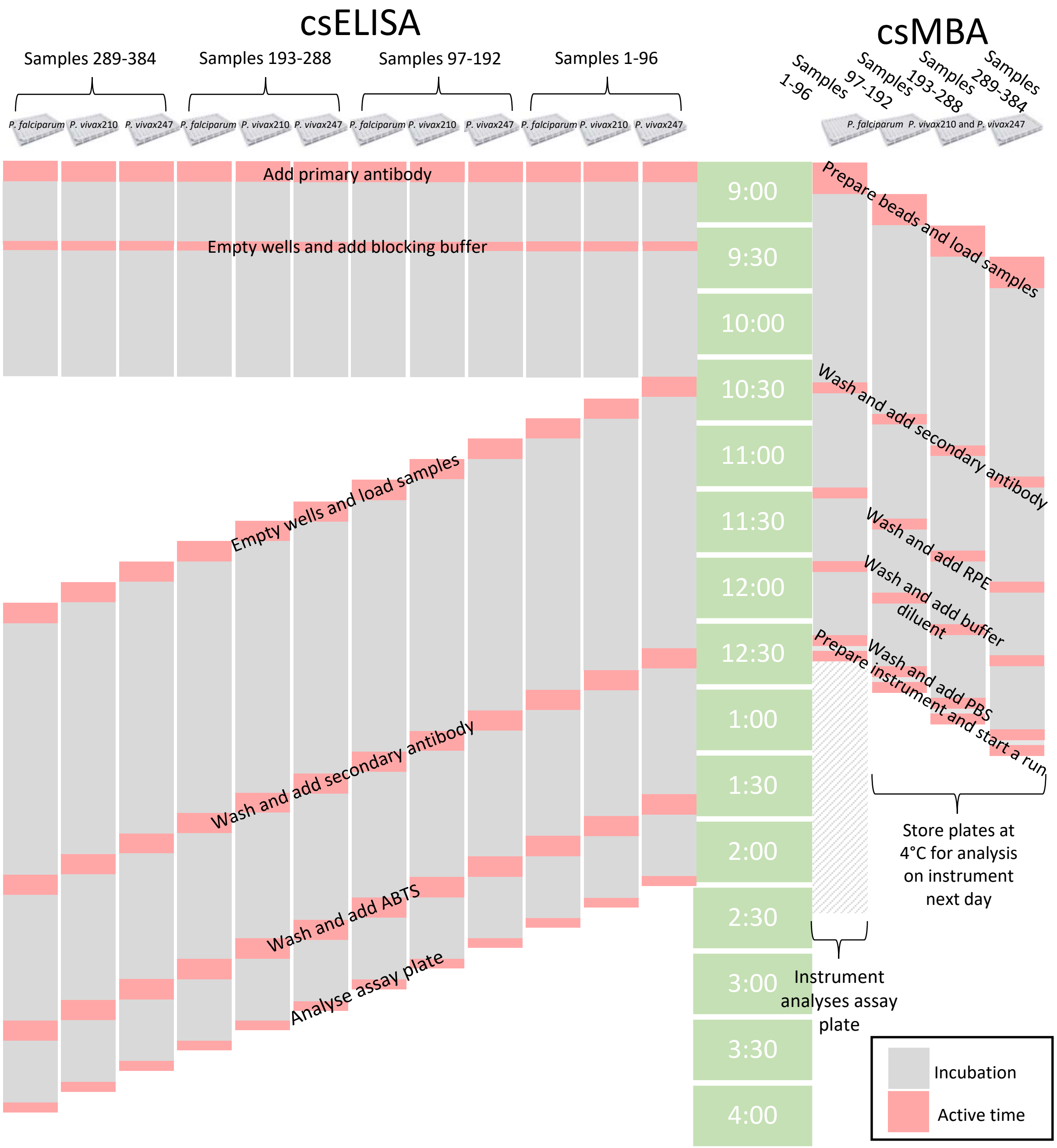

Additional file 2. Example of circumsporozoite (cs) enzyme-linked immunosorbent assay (ELISA) and cs multiplex-bead assay (MBA) workflow for assessing 384 samples with three analytes (*Plasmodium falciparum, P. vivax*210 and *P. vivax*247).
