# Supplementary material for: Adaptation of ELISA detection of Plasmodium falciparum and Plasmodium vivax circumsporozoite proteins in mosquitoes to a multiplex bead-based immunoassay
Source: Malar J. 2021 Sep 23;20:377. doi: 10.1186/s12936-021-03910-z (PMC8461957; doi:10.1186/s12936-021-03910-z)
Supplement: Supplementary file 4 — Additional file 4: Cost analysis of consumables used in the circumsporozoite (cs) enzyme-linked immunosorbent assay (ELISA) and cs multiplex-bead assay (MBA) for assessing three analytes (Plasmodium falciparum, P. vivax210 and P. vivax247) or a single analyte. [file 12936_2021_3910_MOESM4_ESM.pdf]

Assay	Assay step	Item	Example vendor	Example catalog #	Item size	Total cost	Cost per 96-well plate	Cost per mosquito	Cost to assay mosquito for *P. falciparum*, *P. vivax*210 and *P. vivax*247 cs-protein	*Notes*
csELISA	Mosquito preparation	Kontes® grinding tube	Fisher Scientific	05-408-121	500	$63.70	$12.23	$0.13	$0.52	
	P. falciparum csELISA	96-well ELISA plate	Fisher Scientific	07-200-99	100	$288.50	$2.89	$0.03		
		ABTS®2-component peroxidase substrate	Seracare	5120-0033	2.7L	$685.00	$2.54	$0.03		*1350mL each of component A and B. 5mL each component used per 96-well plate = 270 plates per 2.7mL kit*
		200µl pipette tip	Fisher Scientific	02-707-018	960	$70.00	$7.00	$0.07		*One pipette tip per sample*
	*P. vivax*210 csELISA	96-well ELISA plate	Fisher Scientific	07-200-99	100	$288.50	$2.89	$0.03		
		ABTS®2-component peroxidase substrate	Seracare	5120-0033	2.7L	$685.00	$2.54	$0.03		*1350mL each of component A and B. 5mL each component used per 96-well plate = 270 plates per 2.7mL kit*
		200µl pipette tip	Fisher Scientific	02-707-018	960	$70.00	$7.00	$0.07		*One pipette tip per sample*
	*P. vivax*247 csELISA	96-well ELISA plate	Fisher Scientific	07-200-99	100	$288.50	$2.89	$0.03		
		ABTS®2-component peroxidase substrate	Seracare	5120-0033	2.7L	$685.00	$2.54	$0.03		*1350mL each of component A and B. 5mL each component used per 96-well plate = 270 plates per 2.7mL kit*
		200µl pipette tip	Fisher Scientific	02-707-018	960	$70.00	$7.00	$0.07		*One pipette tip per sample*
								Single analyte cost	$0.26	*Mosquito preparation plus P. falciparum, P. vivax210 OR P. vivax247 csELISA*
csMBA	*P. falciparum* csMBA antibody preparation	Bio-Plex™ COOH bead	Bio-Rad	171506020	1mL	$451.00	$4.51	$0.05	$0.71	*Approximately 100 plates per tube of COOH beads at 10µl beads per 96-well plate*
		xMAP® antibody coupling kit	Luminex	40-50016	1 kit	$269.33	$0.67	$0.01		*Kit sufficient for 4 tubes of 1mL microspheres beads. 1mL beads = 100 plates at 10uL beads per 96-well plate*
		EZ-link™ sulfo-NHS-biotin, no-weigh™	Fisher Scientific	PIA39256	10x1mg	$148.00	$0.04	$0.0004		*267.9ng sulfo-NHS-biotin per µl resuspended antibody = 3733µl of antibody labelled per 1mg No-Weigh ™ tube. 10µl labeled antibody per 96-well plate = approx 370 plates*
	*P. vivax*210 csMBA antibody preparation	Bio-Plex™ COOH bead	Bio-Rad	171506018	1mL	$451.00	$4.51	$0.05		*Approximately 100 plates per tube of COOH beads at 10µl beads per 96-well plate*
		xMAP® antibody coupling kit	Luminex	40-50016	1 kit	$269.33	$0.67	$0.01		*Kit sufficient for 4 tubes of 1mL microspheres beads. 1mL beads = 100 plates at 10uL beads per 96-well plate*
		EZ-link™ sulfo-NHS-biotin, no-weigh™	Fisher Scientific	PIA39256	10x1mg	$148.00	$0.04	$0.0004		*267.9ng sulfo-NHS-biotin per µl resuspended antibody = 3733µl of antibody labelled per 1mg No-Weigh ™ tube. 10µl labeled antibody per 96-well plate = approx 370 plates*
	*P. vivax*247 csMBA antibody preparation	Bio-Plex™ COOH bead	Bio-Rad	171506024	1mL	$451.00	$4.51	$0.05		*Approximately 100 plates per tube of COOH beads at 10µl beads per 96-well plate*
		xMAP® antibody coupling kit	Luminex	40-50016	1 kit	$269.33	$0.67	$0.01		*Kit sufficient for 4 tubes of 1mL microspheres beads. 1mL beads = 100 plates at 10uL beads per 96-well plate*
		EZ-link™ sulfo-NHS-biotin, no-weigh™	Fisher Scientific	PIA39256	10x1mg	$148.00	$0.04	$0.0004		*267.9ng sulfo-NHS-biotin per µl resuspended antibody = 3733µl of antibody labelled per 1mg No-Weigh ™ tube. 10µl labeled antibody per 96-well plate = approx 370 plates*
	Mosquito preparation	Kontes® grinding tube	Fisher Scientific	05-408-121	500	$63.70	$12.23	$0.13		
	P. falciparum, *P. vivax*210 and/or *P. vivax*247 csMBA	96-well assay plate	Millipore	MADVN6550	50	$1,140.00	$22.80	$0.24		
		Streptavidin, R-Phycoerythrin conjugate	Fisher Scientific	S866A	1mL	$331.00	$4.97	$0.05		*15 µL per 96-well plate*
		200µl pipette tip	Fisher Scientific	02-707-018	960	$70.00	$7.00	$0.07		*One pipette tip per sample*
		Bio-Plex™ sheath fluid	Bio-Rad	171000055	20L	$75.00	$3.75	$0.04		*Approximately 1L used per 96-well plate*
								Single analyte cost	$0.59	*Mosquito preparation plus P. falciparum, P. vivax210 OR P. vivax247 antibody preparation plus csMBA*

Additional file 3. Cost analysis of consumables used in the circumsporozoite (cs) enzyme-linked immunosorbent assay (ELISA) and cs multiplex-bead assay (MBA) for assessing three analytes (*Plasmodium falciparum*, *P. vivax*210 and *P. vivax*247) or a single analyte.
